# The complete chloroplast genome sequence of *Salix viminalis* (Salicaceae)

**DOI:** 10.1080/23802359.2019.1666041

**Published:** 2019-09-18

**Authors:** Hua-Lei Hu, Hao Lin, Xu-Hui Chen, Yan-Qun Liu, Li Qin

**Affiliations:** a Department of Sericulture, College of Bioscience and Biotechnology, Shenyang Agricultural University, Shenyang, Liaoning, China;; b Department of Bioscience, College of Bioscience and Biotechnology, Shenyang Agricultural University, Shenyang, Liaoning, China

**Keywords:** *Salix viminalis*, chloroplast genome, phylogenetic relationship

## Abstract

Here, the complete chloroplast (cp) genome of *Salix viminalis* was reported. The genome is 155,531 bp long, with a GC content of 36.71%, and contains four sub-regions: 84,395 bp of large single copy (LSC) and 16,218 bp of small single copy (SSC) regions, separated by 27,459 bp of inverted repeat (IR) regions. A total of 129 genes were annotated, including 83 protein-coding genes, 38 tRNA genes, and 8 rRNA genes. The Phylogenetic analyses based on the whole cp genome sequence placed *S. viminalis* into a clade containing *Salix rehderiana*, *Salix taoensis*, *Salix koriyanagi*, and *Salix suchowensis*. This is the first complete cp genome for *S. viminalis* that would be useful for phylogenetic and population genetic studies of this species.

The basket willow *Salix viminalis* is a vigorous, fast-growing shrub willow species and belongs to the genus *Salix*, the largest genus of Salicaceae with 500 species worldwide (Argus 1997). *Salix viminalis* is widely distributed into Europe and Asia. Its most familiar use is for basket-making and fencing. In recent years, it has become familiar as the plant used to absorb heavy metals in contaminated sites and is planted commercially for bio-fuel. We also use it to rear Chinese oak silkworm *Antheraea pernyi*, one of the most known wild silkworms. In this study, we assembled and characterized the complete chloroplast (cp) genome of *S. viminalis* for the first time in order to provide basic genetic information for this important species. The complete cp genome of this species has been deposited into GenBank under accession no. MN117720.

We obtained a specimen of *S. viminalis* from the Silkworm Experimantal Field of Shenyang Agricultural University (N41°50′1.08′′; E123°34′21.92′′), Shenyang, China. The fresh leaves from one single individual were used to extract the total DNA. The specimen was stored with the archival number of SAL_VIM_01 at Department of Sericulture of Shenyang Agricultural University. The genome sequencing was performed using Hiseq 4000 platform by Shanghai Personal Biotechnology Co. Ltd, China. A reference-guided assembly was used to reconstruct the chloroplast genome, with *Salix koriyanagi* (MK120982; Kim et al. 2019) as the reference. The complete cp genome was manually annotated by comparing with the published cp genomes of *Salix* species.

The cp genome of *S. viminalis* is 155,531 bp in length and has a typical circular structure including a pair of inverted repeat (IRs) of 27,459 bp, separated by a large single-copy region (LSC) of 84,395 bp and a small single-copy region (SSC) of 16,218 bp. The overall GC content of *S. viminalis* is 36.71% and in the LSC, SSC, and IR regions are 34.45, 30.97, and 58.13%, respectively. In the cp genome of *S. viminalis*, a total of 129 genes were annotated, of which 110 were unique, consisting of 76 protein-coding genes, 30 tRNA genes, and 4 rRNA genes. Eight protein-coding, seven tRNA, and all four rRNA genes were duplicated in IR regions. In total, 23 intron-containing genes were found, with 4 genes having two introns and 19 genes having one intron.

We then selected 16 Salicaceae cp genomes available, together with *S. viminalis*, to evaluate the phylogenetic position of *S. viminalis* within the *Salix* genus. The phylogenetic tree was reconstructed with MEGA X using maximum-likelihood analysis with GTR + G model (Kumar et al. 2018). The phylogenetic relationships among *Salix* species were supported with moderate to high bootstrap values (Figure 1) and were in line with previous study based on cp genomes where *S. viminalis* was not included (Park et al. 2019). Our phylogenetic analysis placed *S. viminalis* into a clade containing *Salix rehderiana*, *Salix taoensis*, *Salix koriyanagi*, and *Salix suchowensis*, consistent with the study based on morphological characters (Kuzovkina and Volk 2009).

**Figure 1.**
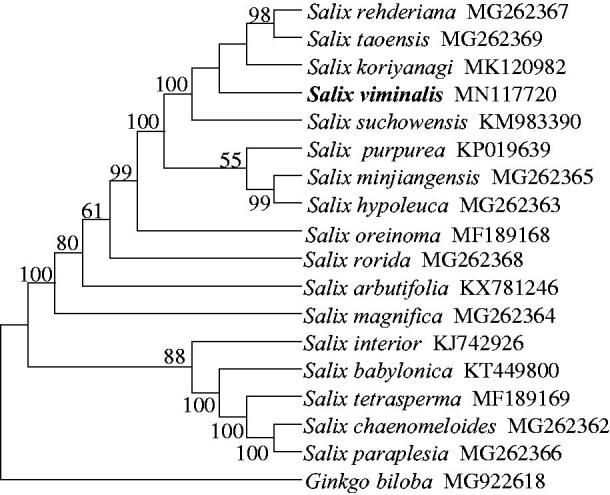
Phylogenetic relationship of 17 *Salix* species built by maximum-likelihood method based on whole chloroplast genomes, with *Ginkgo biloba* as the outgroup. The numbers near branches indicate bootstrap support values.

## References

[CIT0001] Argus GW 1997 Infrageneric classification of *Salix* (Salicaceae) in the new world. Syst Bot Monographs. 52:1–121.

[CIT0002] Kim J, Kim Y, Park J 2019 Complete chloroplast genome sequence of the *Salix koriyanagi* Kimura ex Goerz (Salicaceae). Mitochondrial DNA Part B: Res. 4:549–550.

[CIT0003] Kumar S, Stecher G, Li M, Knyaz C, Tamura K 2018 MEGA X: molecular evolutionary genetics analysis across computing platforms. Mol Biol Evol. 35:1547–1549. 29722887 10.1093/molbev/msy096 PMC5967553

[CIT0004] Kuzovkina YA, Volk TA 2009 The characterization of willow (*Salix* L.) varieties for use in ecological engineering applications: co-ordination of structure, function and antecology. Ecol Eng. 35:1178–1189.

[CIT0005] Park J, Kim Y, Xi H 2019 The complete chloroplast genome sequence of male individual of Korean endemic willow, *Salix koriyanagi* Kimura (Salicaceae). Mitochondrial DNA Part B. 4:1619–1621. 10.1080/23802359.2019.1623115 PMC7687643 33365434

